# Supplementary figures and images for: Field evaluation of the effect of Aspergillus niger on lettuce growth using conventional measurements and a high-throughput phenotyping method based on aerial images
Source: PLoS One. 2022 Sep 19;17(9):e0274731. doi: 10.1371/journal.pone.0274731 (PMC9484672; doi:10.1371/journal.pone.0274731)

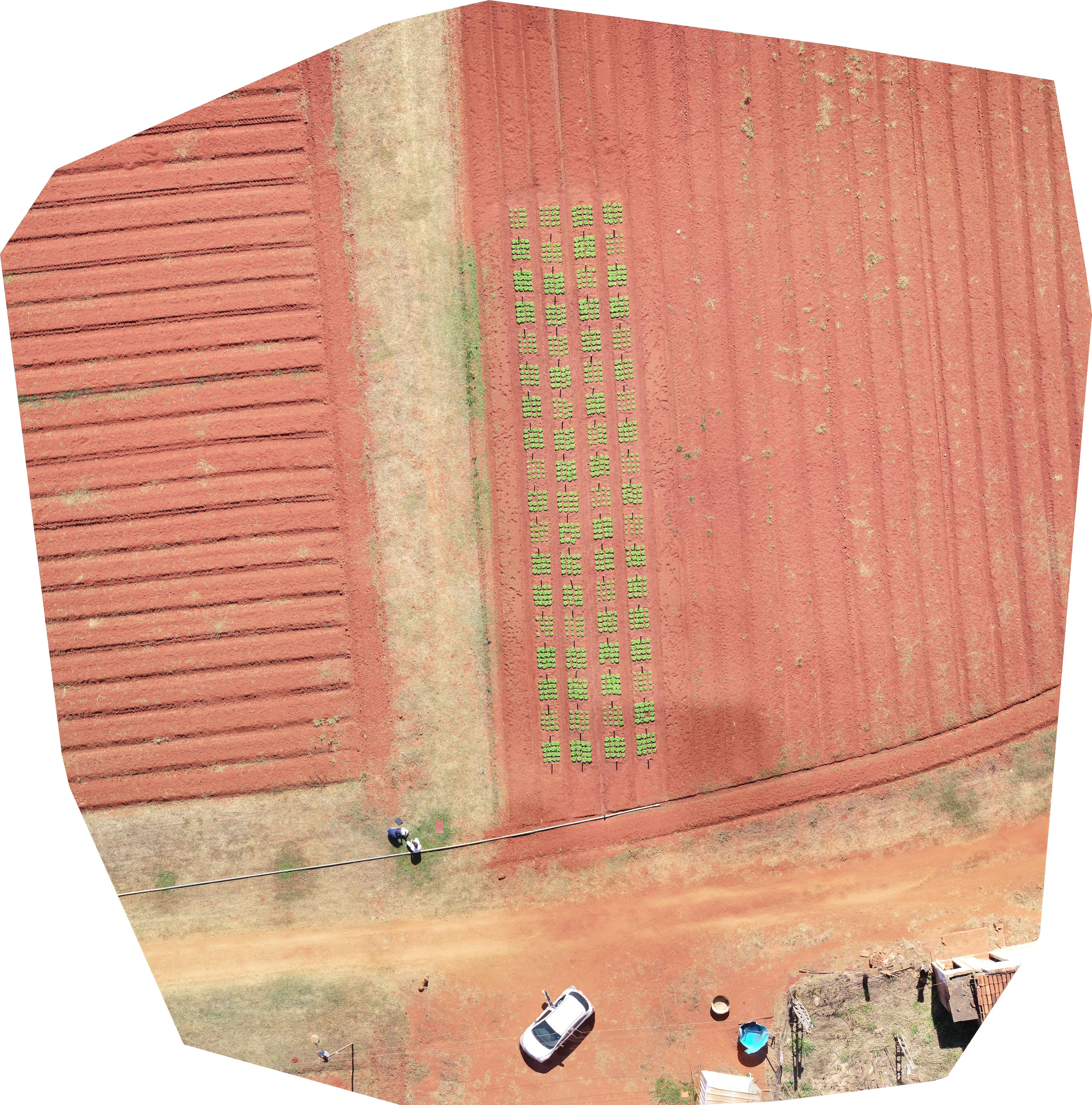

Supplement: S1 Fig — (TIF) [file pone.0274731.s001.tif]
