# Supplementary material for: Field evaluation of the effect of Aspergillus niger on lettuce growth using conventional measurements and a high-throughput phenotyping method based on aerial images
Source: PLoS One. 2022 Sep 19;17(9):e0274731. doi: 10.1371/journal.pone.0274731 (PMC9484672; doi:10.1371/journal.pone.0274731)
Supplement: Attachment — Submitted filename: Response to reviewers.docx [file pone.0274731.s002.docx]

Dear Dr. Ali Tan Kee Zuan

Academic Editor

We are pleased to resubmit the manuscript PONE-D-22-21385.

We are thankful for the time dedicated to our work. We have reviewed carefully the issues raised by reviewers. Following are the responses to their comments. All changes are highlighted in the manuscript.

We hope this new version is acceptable for publication.

Best regards,

Gilberto O. Mendes

Point-by-point response to comments:

**ACADEMIC EDITOR**

Both reviewers have agreed that the title of the manuscript should be improved to reflect the content.

Some minor amendments are needed as indicated by both reviewers.

**Authors:** As suggested by reviewers, we changed the title to better fit the manuscript content. We also corrected all minor issues indicated by reviewers.

**Reviewer #1**

In my opinion, the title of this manuscript should be changed to give more focus and emphasis on high-throughput phenotyping methods based on aerial images to determine the effect of microorganism inoculation on plant growth. The technique has potential for large-scale cropland surveys.

**Authors:** We agree with you. We changed the title to give more emphasis on the phenotyping method based on aerial images.

Line 141: Sample of the orthoimage in Figure 1 is missing.

**Authors:** Sorry for the mistake. We included Fig 1 and hence renumbered the following figures.

Line 263-265: The authors discuss the switch mechanisms of beneficial traits of inoculum and the host plant’s needs. A complete discussion of the mechanisms should be included in the manuscript (discussion section).

**Authors:** We improved the discussion at this point, including more details about the mechanisms. See lines 269-273.

Line 302-307: Conclusion should be rewritten with additional emphasis on the potential of aerial imaging techniques to determine the effect of A. niger on growth of lettuce. In addition, the theory about switch mechanisms related to bacterial potency and the need of lettuce (host plants) needs to be explained in depth.

**Authors:** Conclusive paragraph was rewritten as suggested (lines 311-318). The theory about switching mechanisms was better discussed as well (lines 269-273).

**Reviewer #2:**

Title ambiguous, need to change, not reflected with objective

**Authors:** We changed the title to better fit the manuscript content.

line 38-39, sentence to rephrase or some grammatical errors

**Authors:** Rephrased. See lines 39-40.

line 46, ...diminish the gap in the crop yield potential.

**Authors:** Corrected. See line 47.

line 47, to improve sentence.

**Authors:** Sentence was rephrased. See lines 48-49.

line 115, by pipetting 3 mL per tray cell 30 min before planting. Need explain, not clear, how do you do this?

**Authors:** Text rephrased for clarity. See lines 115-117.

line 123-124, explain in detail, approx. size of one granule. how to direct contact with the seedling root?

**Authors:** We included a description of the shape of granules. Also, we have clarified the way we applied the granules to the seedling. See lines 124-126.

Figures low resolution, improve it.

**Authors:** We have reprocessed all figures using the PACE tool according to the PLOS guidelines.
